# Detection and assessment of dust mite allergens in an indoor environment in Anhui, China

**DOI:** 10.1007/s11356-022-22395-0

**Published:** 2022-08-08

**Authors:** Qiqi Xue, Minghui Zou, Junjie Guo, Qiao Teng, Qiqi Zhang, Lingwei Sheng, Sijia Xu, Can Fang, Ning Yao, Yuanyuan Li, Jinhong Zhao

**Affiliations:** 1 Department of Medical Parasitology, Wannan Medical College, WuhuAnhui, 241002 China; 2 Department of Medical Parasitology, Qiqihar Medical College, QiqiharHeilongjiang, 161000 China; 3 School of Clinical Medicine, Wannan Medical College, WuhuAnhui, 241002 China; 4 Medical Laboratory Science, Wannan Medical College, WuhuAnhui, 241002 China; 5 School of Public Health, Wannan Medical College, WuhuAnhui, 241002 China; 6 Anhui Province Key Laboratory of Biological Macromlecules Research, Wannan Medical College, WuhuAnhui, 241002 China

**Keywords:** Dust mite, Air conditioner dust, Floor dust, ELISA questionnaire

## Abstract

Dust in the home environment is thought to be a potential trigger for increasing allergic diseases, such as allergic rash, rhinitis, asthma, and other conditions, associated with dust mites. To verify the status of dust mite prevalence in indoor surroundings, we collected 189 dust samples from the air conditioner filters (*n* = 75) and floors (*n* = 114) of households, schools, and hotels in the Anhui area, China. All samples were measured for dust mite breeding rate and breeding density under light microscopy and analyzed for dust mite species *Dermatophagoides farinae* 1 (Der f 1) and *Dermatophagoides pteronyssinus* 1 (Der p 1) allergen using enzyme-linked immunosorbent assay (ELISA). The dust mite breeding rates were 34.67% (26/75) and 20.18% (23/114), respectively, in the dust samples from the floor and air conditioning filters. The breeding density was the highest in households (10/g), followed by schools (9/g) and hotels (4/g). ELISA indicated that the allergen threshold (2.0 µg/g dust) of Der f 1 was exceeded in only two samples and Der p 1 in one sample. Additionally, a questionnaire was used to investigate the health knowledge on allergic diseases involved in indoor facilities, finding that most allergy sufferers were aware that indoor dust might be responsible for their conditions. The findings suggest that regular maintenance of indoor hygiene and cleaning of air-conditioning filters should reduce the risks of exposure to indoor allergens.

## Introduction

Mite allergens belong to the group of inhalant allergens and represent antigenic substances that are particularly important in the pathogenesis of respiratory system diseases and skin diseases (Chermprapai & Thengchaisri 2020). The most common diseases associated with chronic exposure to these aeroallergens include allergic rhinitis, bronchial asthma, and atopic dermatitis (Siwak et al. 2014) . Bodies, feces, eggs, and corpse residues of mites can cause severe allergic reactions. Thus, when mites multiply in large numbers in the human living environment, they easily make contact with human skin and enter the respiratory tract, causing allergic reactions (Wilson & Platts-Mills 2018). The dominant allergens of the most common house dust mite species (*Dermatophagoides pteronyssinus* and *Dermatophagoides farinae)* belong to mite Group 1 (Der p 1 and Der f 1) (Manuyakorn, et al. 2015; Pomes et al. 2016).

People spend most of their time indoors, especially during the hot seasons. This will result in considerable adverse health effects due to long exposure to indoor pollutants (Jacobs et al. 2014) . Most patients with asthma and allergic disease are extremely sensitive to indoor allergens related to dust mites, animal fur, insects, and fungal spores (Agache et al. 2019; Borchers et al., 2017; Matricardi et al. 2016; Moote et al., 2018; Schuler Iv & Montejo, 2019). Exposure to high levels of dust mite allergens can exacerbate asthma and lead to other related conditions such as allergic rhinitis and conjunctivitis, atopic dermatitis, and urticaria (Alba et al. 2010) . *Dermatophagoides pteronyssinus* and *Dermatophagoides farinae* are the main species of dust mites that are distributed worldwide and are the most commonly inhaled indoor allergens. House dust mites (HDMs) are a primary allergen worldwide, and preceding studies conducted to explore this have proven that more than 90% of allergic rhinitis (AR) patients in central China have been triggered with the aid of HDMs (Wang et al., 2017). A multivariate logistic regression analysis conducted by Gasana et al. (Gasana et al. 2021) for the prevalence and severity of allergic diseases arising from indoor exposure to dust mites in American households demonstrated that the presence of dust Der f 1 was responsible for asthma and/or severe wheezing. Therefore, understanding the indoor transmission mode and concentration distribution of dust mite allergens is essential to investigation of the pathogenesis of allergic diseases. Thus, we accumulated dust samples from extraordinary indoor environments and examined the morphology of dust mites and the concentrations of allergens Der f 1 and Der p 1.

Dust mites are highly ubiquitous in the dust of indoor spaces and lodged with dust descending on floors, mattresses, carpets, and corners (Zare et al. 2021) . Apart from that, dust cannot be ignored in indoor air conditioning filters, in which cotton fibers and fungal spores are densely accumulated, providing a favorable condition for mite breeding. In recent years, some reports have indicated that air conditioning filter dust can cause allergic diseases (Arroyave et al. 2013; Ravindra 2019; Ravindra & Smith 2018). Khaliq et al. described the allergic symptoms of breathing difficulties, infection in the upper respiratory tract, dryness, redness on the skin, headache, drowsiness, and watery eyes in customers associated with air conditioner filters (Khaliq et al. 2006). However, few studies are available on the influence of allergen concentration in dust from air conditioning filters. Therefore, in addition to indoor floor dust, accumulated dust from air conditioning filters was used as research and objective in this study.

Environmental epidemiology is an indispensable means to assess pollutant exposure in the environment and human health (Dedele et al. 2019). This study was designed to determine the breeding rate and density, morphology, and allergen of dust mites in house dust and air condition filters in Anhui, China. To better understand why the breeding rate of dust mites varied at dissimilar settings, we further examined the relationship between dust mite allergens and house characteristics and meteorological parameters in house dust and air condition filters. These findings may be conducive to developing rational preventive measures to reduce dust mite allergen exposure and provide a theoretical basis for the prevention and treatment of dust mite allergies.

## Materials and methods

### Sampling sites

Anhui is an inland province in Southeast China, stretching over two climatic zones between the subtropics and warm temperate that are featured by the Huaihe River. Cities south of the Huaihe River are warm and humid, while cities north of the Huaihe River are relatively dry and cooler. There are 12 cities in Anhui (Table 1), of which 7 are situated in the southern region of the Huaihe River, with a subtropical monsoon climate and ample rainfall. Therefore, this study was designed by cross-sectioning of the dust samples from indoor floors and air conditioning filters in civilian residences, schools, and hotels across the north and south Huaihe areas in Anhui Province of China.

**Table 1:** Samples collected in this study

City location	Dust sample (*N* = 189)			Climate character
	Air conditioning filters (*n* = 75)	Floor (*n* = 114)	Positive samples (*n*, %)	
**To the south of Huaihe River**	**44**	**62**	**35 (18.52%)**	
Hefei	6	9	5 (33.33%)	Subtropical monsoon climate
Wuhu	7	8	6 (40%)	Subtropical humid monsoon climate
Lu’an	7	10	5 (29.41%)	Subtropical monsoon climate
Chuzhou	6	8	3 (21.43%)	Subtropical monsoon climate
Xuancheng	6	9	5 (33.33%)	Subtropical monsoon climate
Anqing	5	8	4(30.77%)	North subtropical humid monsoon climate
Huainan	7	10	7 (41.18%)	Subtropical monsoon climate
**To the north of Huaihe River**	**31**	**52**	**14 (16.87%)**	
Bengbu	7	10	3 (17.65%)	A transitional climate from subtropical monsoon climate to temperate monsoon climate
Huaibei	5	8	2 (15.38%)	Temperate monsoon climate
Suzhou	6	12	3 (16.67%)	Temperate monsoon climate
Fuyang	7	13	4 (20%)	Temperate monsoon climate
Bozhou	6	9	2 (13.33%)	Temperate monsoon climate

### Dust sample collection

The dust was generally collected from the floors and air conditioning filters at each collection site. Floor dust samples were harvested from four different areas in each private dwelling, including the bedroom, living room, mattress, and upholstered furniture. Air conditioning filters were initially removed, and then the dust was gently swept with a brush. All research assistants underwent training on the sampling procedures before sample collection. To remove particles that were greater than 425 μm in diameter, the dust samples collected from floors were passed through a metal sieve with 50 meshes (Liu et al. 2007) . The dust from air conditioning filters was initially treated by removal of the visible large flocculent residues using a clamp, with flocculent dust being left for subsequent experiments. All sampling sites were measured for the indoor temperature and relative humidity (RH), and all dust samples were sealed on-site in individual sampling process and brought back to the lab for subsequent study.

### Measurement of the dust mites

Each dust sample was transferred into a sterilized petri dish and microscopically counted and identified for the mite species. All the samples were weighed three times using a microbalance, and the mean weight was recorded. The breeding density and morphology of dust mites were measured and observed under a light microscope. Morphological examination and identification of dust mites were based on their body characteristics according to previous descriptions (Hughes 1976; Li et al. 2014) .

### Determination of Der f 1 and Der p 1 allergen concentrations

Der f 1 and Der p 1 allergens were extracted by suspending 100 mg dust in 2 mL phosphate-buffered saline with 0.05% Tween-20 (PBS-T). After ultrasonic grinding for 5 min, the solution was placed in a refrigerated track oscillator (Thermo Scientific™ MaxQ™ 6000) at 4 °C and 100 r/min for 2 h and then centrifuged at 3000 g at 4 °C for 15 min. Allergen levels were detected using a two‐site monoclonal antibody‐based enzyme‐linked immunosorbent assay with a Der f 1 and Der p 1 ELISA 2.0 kit (INDOOR, Biotechnologies, Ltd, Manchester UK). Next, the sample extracts (100 µL) were added in triplicate into each plate, and a 10‐point curve was made using sequential twofold dilutions of a known standard in PBS‐T with 1% BSA (Vesper et al. 2016) . Finally, the samples were read with an optical density plate reader at a wavelength of 450 nm. The limit of detection (LOD) of the sample was set at 0.19 ng/mL for Der f 1 and 0.78 ng/mL for Der p 1.

### Questionnaire survey

The research group obtained written informed consent and conducted corresponding surveys using a 12‐item questionnaire upon household sampling. The survey consisted of gender, age, characteristics of sampling sites, individual history of allergies and family history of allergies, and health assessment.

### Statistical analysis

Data analysis was performed using SPSS statistics 21.0 software (SPSS Inc., Chicago, IL, USA). Descriptive statistics (geometric mean, median, geometric standard deviation, and percentiles) were used to characterize the data. The prevalence of dust mite allergies was measured in percentages, and the chi-square test was used for categorical variables. A chi-square test was performed on the breeding rates of dust mites in different collection locations, floors, room orientations, cleaning frequencies, and ventilation times, and their statistical significance was compared.

*Ethical statement.*

All components of the study were carried out in accordance with national ethics rules and authorized through the Medical Ethics Committee of Wannan Medical College. Informed written consent was also obtained from the eligible participating individuals before the interviews.

## Results

### Characteristics of sampling sites

In total, 189 specimens were obtained from 105 homes, of which 45 were from hotels; 82 were from residential buildings; and 62 were from school dormitories. The sampling sites covered 12 cities in the south and north of the Huaihe River in Anhui, China. The specific sample numbers are shown in Table 1. A total of 39.78% of the samples were collected from rooms located in buildings with fewer than three stories. A total of 54.3% of the rooms were between four and ten stories, and 15.59% were above ten stories. As to the length of sunshine exposure and ventilation of the rooms where we collected samples, nearly half (49.46%) of them were facing south, 37.63% facing north, and the remaining 5.38% and 3.76% facing east and west, respectively. In addition, our sampling was done in July and August, which were the hot seasons in the Anhui area, and therefore the air conditioning system was running in the houses we sampled, yet the frequency of air conditioner use and ventilation time varied for each sampling room. The cleaning frequency of air conditioning filters and the cleaning cycle of bedroom mattress were also different at different sampling sites (Table 2).

**Table 2:** Characteristics of sample collection sites

Characteristics	Dust samples, *n* (%)	Breeding rate	Breeding density (n/g)			
		**Positive samples, *n* (%)**	***P-*value**	**GM**	**Min**	**Max**
Air conditioning dust						
Sample sites			> 0.05			
Total	75	26 (34.67%)		2.04	0	10
Household	32 (42.67%)	10 (31.25%)		2.22	0	10
School	23 (30.67%)	12 (52.17%)		1.91	0	6
Hotel	20 (26.67%)	4 (20%)		2	0	4
Floors			< 0.01			
Less than 3 floors	29 (38.67%)	18 (62.07%)		2.04	0	10
4–9 floors	41 (54.67%)	7 (17.07%)		2.25	0	4
More than 10 floors	5 (6.67%)	1 (20%)		1	0	1
Room orientation			< 0.05			
East	2 (0.27%)	0		0	0	0
South	39 (52%)	8 (20.51)		1.77	0	4
West	3 (4%)	1 (33.33%)		1	0	1
North	31 (41.33%)	17 (54.84%)		2.27	0	10
Cleaning frequency of air conditioning filters			< 0.01			
Less than 1 month	2 (2.67%)	0		0	0	0
1–3 months	18 (24%)	2 (11.11%)		1	0	1
4–6 months	23 (30.67%)	6 (26.09%)		2	0	4
More than 6 months	32 (42.67%)	18 (56.25%)		2.22	0	10
Indoor ventilation time			< 0.01			
Less than 30 min	0					
30 min to 1 h	12 (16%)	10 (83.33%)		2.67	0	6
1 h to 3 h	21 (28%)	10 (47.62%)		1.99	0	10
More than 3 h	42 (56%)	6 (14.29%)		1.35	0	3
Floor dust						
Sample sites			> 0.05			
Total	114	23 (20.18%)		2.67	0	9
Household	50 (43.86%)	12 (24%)		2.71	0	6
School	39 (34.21%)	8 (20.51)		2.91	0	9
Hotel	25 (21.93%)	3 (12%)		2	0	4
Floors			< 0.01			
Less than 3 floors	46 (40.35%)	15 (32.61%)		2.75	0	9
4–9 floors	59 (51.75%)	5 (8.47%)		2.95	0	6
More than 10 floors	9 (7.89%)	3 (33.33%)		1.82	0	3
Room towards			< 0.05			
East	3 (2.63%)	0			0	0
South	58 (50.88%)	6 (10.34%)		2.26	0	4
West	7 (6.14%)	2 (28.57%)		3.16	0	5
North	46 (40.35%)	15 (32.61%)		3.84	0	9
Cleaning frequency of bedroom mattress			> 0.05			
Less than 1 week	56 (49.12%)	8 (14.29%)		1.77	0	3
2 to 4 weeks	38 (33.33%)	8 (21.05%)		3.02	0	6
5 to 8 weeks	18 (15.79%)	6 (33.33%)		3.85	0	9
More than 8 weeks	2 (1.75%)	1 (50%)		3	0	3
Indoor ventilation time			> 0.05			
Less than 30 min	0					
30 min to 1 h	19 (16.67%)	4 (21.05%)		3.83	0	9
1 to 3 h	31 (27.19%)	5 (16.13%)		2.35	0	6
More than 3 h	64 (56.14%)	14 (21.88%)		2.52	0	5

*GM* geometric mean, *Min* minimum, *Max* maximum

### Characteristics of dust samples

Overall, 189 dust samples were harvested from July through August 2021. Seventy-five samples were from air conditioning filters, and 114 were from indoor floors (Table 2). However, most of the dust samples were collected from both floors and air conditioning filters in the same space. Only 39 samples were obtained from rooms where no air conditioner or the air conditioning filters had been cleaned recently.

### Morphological examination of dust mites

**Fig. 1:**
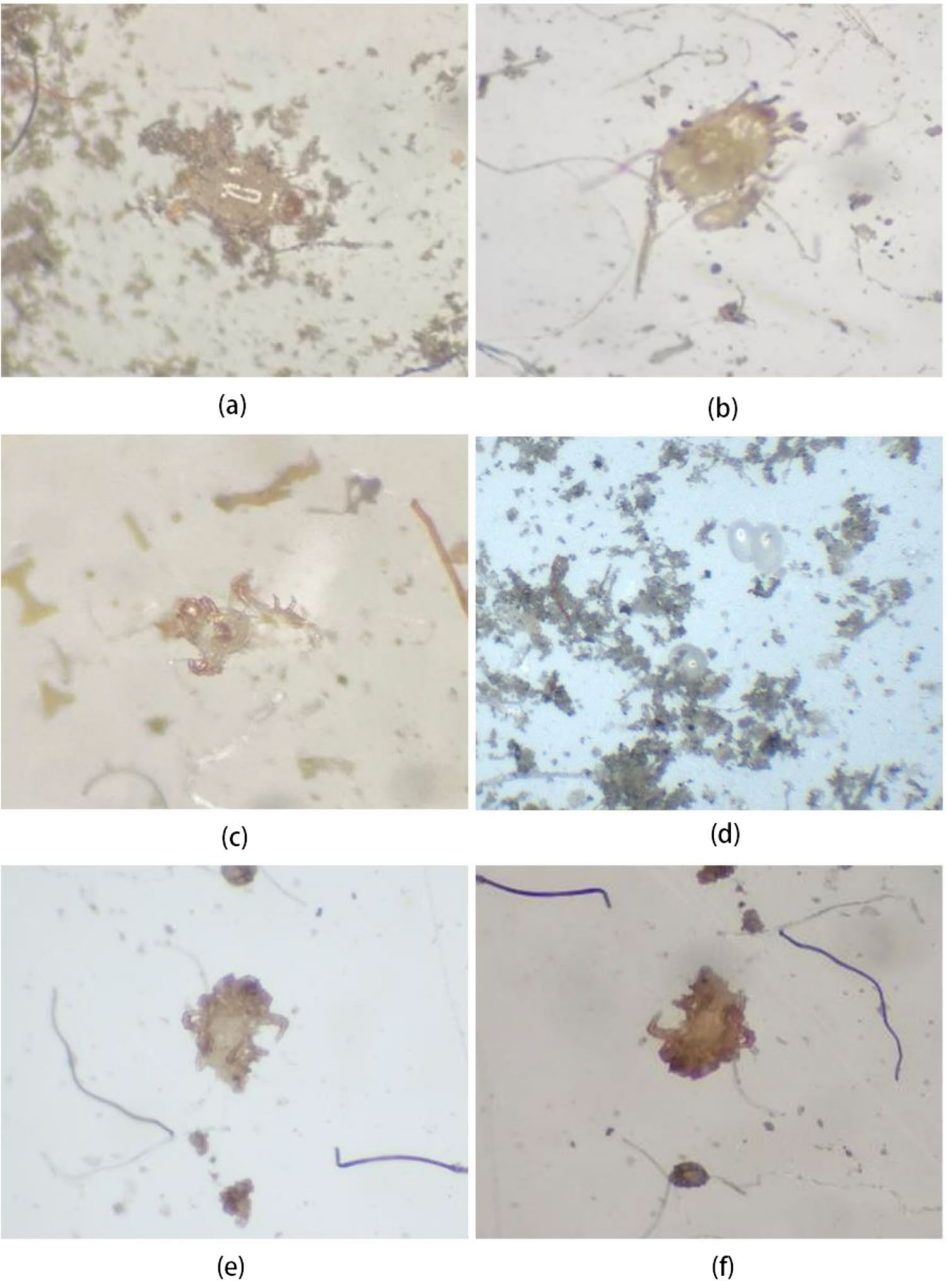
Microscopic examination of dust mites: (a) dust mite in air conditioning filter; (b) adult and larva mites in floor dust; (c) broken adult mite; (d) mite eggs in dust; (e) shrivelled mite body; (f) crouched and shrivelled mite body

The mite species in dust samples were preliminarily observed using an optical microscope. Most of them were identified as *Dermatophagoides* and *Acarus* (K. Sharma et al. 2019; van Boven et al., 2020a, b). Some living mites were obtained (Fig. 1a, b) and made slide specimens that were observed under a light microscope for body characteristics, including gnathosoma, seta, and solenidia. Meanwhile, many remnants of withered broken mite bodies were found in the dust samples (Fig. 1c, f), making it difficult to identify the mite species. Some mites became dehydrated and shriveled after death, leaving only parts of limbs (Fig. 1c, e, f). Additionally, numerous eggs of dust mites were detected in the dust samples. These findings showed that this dust can provide an appropriate living environment for dust mites to breed, and there, dust mites can proceed to grow, develop and reproduce (Fig. 1d).

### Breeding rate and breeding density of the dust mites

The breeding density of dust mites was microscopically verified in 189 dust samples collected from different locations. A total of 49 specimens were found to contain dust mites. The geometric mean (GM) breeding density of all positive specimens was 2.31/g. The highest breeding density was observed for samples from households (10/g), schools (9/g), and hotels (4/g). In the 75 samples from air conditioning filters, the breeding density GM was 2.04/g, and the maximum breeding density was 10/g. The GM breeding density of positive dust mites in the 114 floor dust samples was 2.67/g, and the maximum breeding density was 9/g.

Microscopical examination revealed that 35 (32.41%) dust samples were positive for dust mites in the 108 specimens collected from cities south of the Huaihe River, whereas only 14 (16.87%) of the 83 samples collected from areas north of the Huaihe River were positive for dust mites (Table 1). The difference was significant by the Huaihe River as a boundary (*p* < 0.05). Of the 75 dust samples from air conditioning filters, the total number of positive specimens was 26 (34.67%), and 23 (20.18%) of the 114 floor dust samples appeared positive for dust mites. In addition, the detection rate varied to a certain degree among the samples harvested from three different sites, which represented 26.83% (22/82) in house dust, 32.26% (20/62) in school dormitories, and 15.56% (7/45) in hotel rooms.

**Fig. 2:**
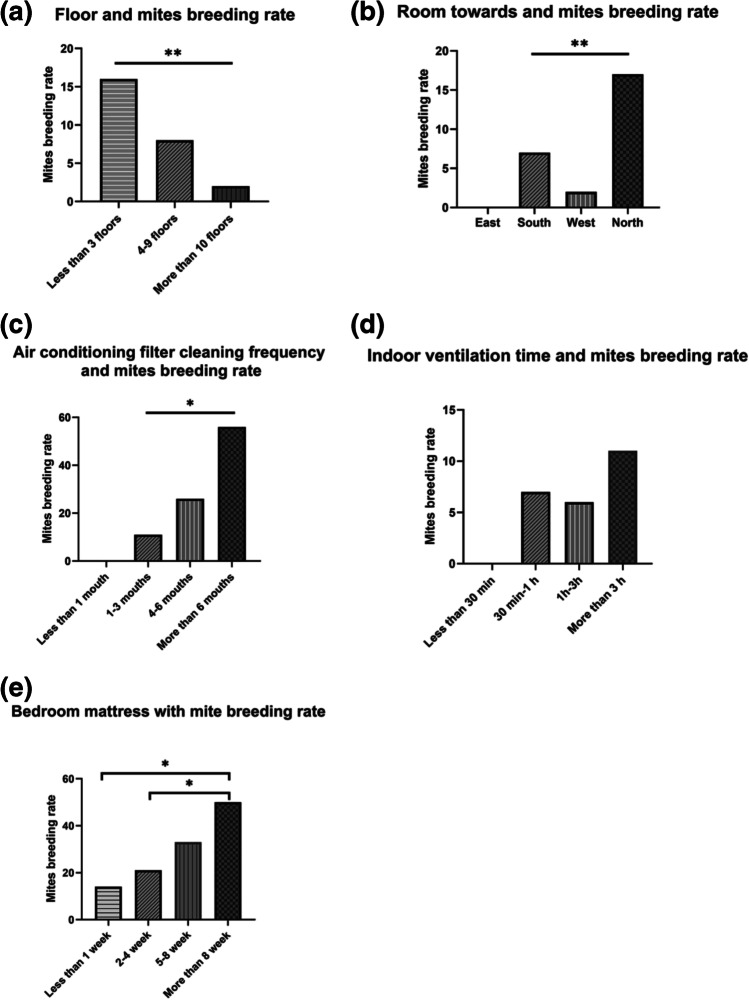
Relationship between mite breeding rate and different room parameters. (a) The relation between mite breeding rate with different floors; (b) the relationship between room orientation and mite breeding rate; (c) the relationship between cleaning frequency of air conditioning filter with mite breeding rate; (d) the relationship between indoor ventilation duration with mite breeding rate; (e) The relationship between bedroom mattress with mite breeding rate. *p < .05, **p < .01; * Statistical differences between the two groups of data *p* < . 05

Regarding the nature of the sampling sites, the breeding rate of mites was diverse as well. The breeding rate presented a negative correlation in the room floor samples (Fig. 2a), and there were significant differences among the cleaning frequencies of air conditioning filters (Fig. 2c) (*p* < 0.01) and bedroom mattresses (Fig. 2e) and the time of indoor ventilation (Fig. 2d) (*p* < 0.05). The difference was also significant in cleaning frequencies of bedroom mattresses of less than 1 week, more than 8 weeks, 2–4 weeks, and more than 8 weeks. Furthermore, room orientation (Fig. 2b) was associated with dust mite detection rate; for instance, the breeding rate was 37.66% (29/77) in the dusts from rooms facing north and 17.53% (17/97) from those facing south; the former was significantly higher than the latter (Table 2).

### Measurement of Der f 1 and Der p 1 allergen concentration

According to the LOD for Der f 1 (0.19 ng/mL of sample) and Der p 1 assay (0.78 ng/mL), Der f 1 allergen was detected in 61 samples of floor dusts, with a detection rate of 53.5% (61/114). Der p 1 allergen was found in 49 floor dust samples, with a detection rate of 42.98% (49/114). However, the detection rates of Der f 1 and Der p 1 allergens were 32% (24/75) and 20% (15/75), respectively, for the dust samples from air conditioning filters. Nevertheless, clinically significant sensitization by Der f 1 and Der p 1 allergen concentrations (> 2.0 µg/g dust) (Cao & Liu 2020; “Dust mite allergens and asthma: a worldwide problem. International Workshop report,” 1988; Nitschke et al. 2006) was found only in two families and one family, respectively. These samples were obtained from the bedroom carpet and bedroom floor, with the concentration of Der f 1 allergen at 2.23 μg/g dust and 2.08 μg/g dust, and Der p 1 allergen at 2.19 μg/g dust. Although the concentrations of Der f 1 and Der p 1 allergens detected in most of our samples did not satisfy clinical allergy criteria, our results were similar to those of a previous report on the detection rate of dust mite allergens in household dust (Johnston et al. 2019).

### Analysis of the questionnaire survey

#### Allergy history and family history

Of the total study participants, 40 (18.96%) reported allergic diseases, whereas 171 (81.04%) had no known allergies. Those who reported allergies also had family a history of rhinitis (35%), asthma (7.5%) and recurrent urticaria (12.5%) (Table 3), yet a family history of these diseases was free in approximately 45% of the allergic respondents.

**Table 3:** Percentage of respondents with allergic family history

Family history	Allergic (*N* = 211)	
	**Yes**	**No**
**Rhinitis**	14 (35%)	6 (3.50%)
**Recurrent urticaria**	5 (12.50%)	4 (2.34%)
**Asthma**	3 (7.50%)	0 (0%)
**None**	18 (45%)	161 (94.15%)

#### Analysis of air conditioner use

The subjects who completed the questionnaire survey were between 15 and 60 years old. According to the survey results, 37% of interviewees spent 6–12 and 33% spent 3–6 h in air conditioned rooms. Another 24% stayed less than 3 h. Only 6% of them spend more than 12 h in the air conditioned space. Among all interviewees, more than half (69%) had a habit of cleaning the air conditioning system every 6 months or so; 25% cleaned it in an interval of 3 months; and less than 6% replaced the air filter once a month.

#### Self-reported allergy trigger

Most study participants (65%) reported that their allergies were triggered by indoor dust. Only a small number of them (5%) had ever had a test for the diagnosis of the allergen associated with pollen, fish, shrimp, pet hair, etc. However, 30% of respondents were not sure what triggered their allergies. The known and unknown triggering factors for the allergic conditions by the respondents are illustrated in Table 4.

**Table 4:** Percentage of triggering factors in respondents with allergic reactions

Triggering factor	Allergic response (*N* = 211)	
	**Yes**	**No**
**House dust**	38 (95%)	99 (57.89%)
**Pollen**	2 (5%)	4 (2.34%)
**Others**	0 (0%)	2 (1.17%)
**None**	0 (0%)	66 (38.60%)

Among those with allergies, some were aware that the use of the air conditioner had an effect on their susceptibility to allergies. Therefore, we hypothesized that the questionnaire respondents with allergic reactions should be involved in the use of air conditioners.

## Discussion

Asthma and allergic sensitization have become increasingly prevalent in recent decades and have resulted in a significant impact on the economy and quality of life of victims as well as an urgent understanding of the potential causes for effective prevention (Huang et al. 2020) . House dust mites are arguably the masters of allergenicity worldwide. Atopic reactivity to their products is one of the most common causes of allergies and affects the eyes, upper and lower airways, skin, and, on occasion, systemic circulation (Calderon et al. 2015; Sanchez-Borges et al. 2017) . Exposure to microbes and allergens in people with an allergic constitution and at different exposure levels can exacerbate allergic conditions in the course of diseases (Wilson & Platts-Mills, 2018). The home is a key microenvironment for exposure to allergens, yet children and adults are estimated to spend an average of 60 to 80% of their time indoors, since continuous exposure, even to low levels of allergens, in individuals with mild asthma may exacerbate symptoms and bronchial hyperresponsiveness (Van Boven et al. 2020a) . Therefore, it is critical to evaluate the allergen level of dust mites in the indoor environment for the prevention and treatment of allergic diseases.

This study investigated the dust mite density and allergen concentration in indoor dust collected from floors and air conditioning filters in Anhui Province, China. All samples were comprehensively assessed by microscopy and tested for the allergen concentration in the samples. In addition, a questionnaire survey was performed to understand the association of indoor dust exposure with allergic conditions in subjects whose rooms were sampled. Dust mites were generally detected in the dust samples from floors and air conditioning filters. Microscopical and morphological identification showed that the mites in our samples were the most common species of *Dermatophagoides* and *Acarus* and revealed intact young mites, adult mites, and broken limbs in the dust samples. The findings suggest that dust mites are prevalent in indoor environments, which is consistent with a previous report (K. Sharma et al. 2019). Suitable temperature and humidity are considered to be favorable to the growth and development of mites. Sharma et al. described that dust mite allergens were detected in dust samples from air conditioning filters when the temperature was between 18 and 23 °C, and the humidity was between 47 and 59% (Sharma et al. 2019) . Similar results were also reported by Matsui et al. (Matsui et al. 2008) who found that a higher level of allergen was associated with increased moisture. This evidence further demonstrates that accumulated dust in the air conditioning filters along with the temperature and humidity tends to create an appropriate environment for mite breeding and allergen concentration indoors.

In our observation, the breeding rate of dust mites was significantly higher in the dust samples from the air conditioning system than in those from the floors (34.67%, 26/75; vs. 20.18%, 23/114). We considered that the dust on the air conditioning filter screen was more concentrated and stable than the indoor floor dust and less affected by natural ventilation; in particular, running the air conditioner in summer can ensure stable temperature and humidity indoors, which is more conducive to the breeding and reproduction of dust mite microbes (Prasad et al. 2009) . In a similar study, Liu et al. (Liu, et al., 2007) reported house dust mites in air conditioning filters in Shenzhen city, China. The study identified various species of dust mites including *Dermatophagoides farinae*, *Dermatophagoides pteronyssinus*, *Cheyletus trouessarti*, *Cheyletus malaccensis*, *Spinibdella* sp., *Blomia freeman*, and *Lepidoglyphus destructor*, and reported the highest population in July and August. Sharma et al. (Sharma et al. 2011) determined that indoor dust mites in suspected patients with a history of allergic disease were monitored for *Dermatophagoides*, *Blomia*, *Acarus*, and *Cheyletus* species. Additionally, the dust housed in the air conditioning screens generally presents as floccus, which is more difficult to detect under the microscope than ordinary floor dust. Therefore, the actual breeding density of dust mites in the air conditioning filters should be higher. By comparison among the three different sampling places, the detection rate was obviously lower in hotel rooms than in households and schools. This may be the consequence of the regular and relatively higher cleaning frequencies of the air conditioning system in hotels.

The breeding rates of dust mites varied to a certain degree in different geographical locations, with higher rates in cities located in the south than in those located in the north of the Huaihe River. We also observed that the detection rate was the highest in dust samples obtained from rooms under 3 floors, where the detection rate was 40% and the density of dust mites was 10 mites/g. This must be associated with poorer sunshine exposure and closer proximity to the ground of the rooms that are much darker and damper, which may provide better humidity conditions for the breeding of mites. In addition, most rooms in China face either north or south. Rooms facing south are generally warmer and drier than those facing north because of better sunshine exposure and natural ventilation. This is why we observed a significantly higher dust mite breeding rate in the north-facing rooms than in the south-facing rooms. Another explanation is that humidity from the air conditioning system may provide enough moisture to indoor air to allow the survival of mites in homes in dry seasons (Wu et al. 2009) . Similar results were reported by Matsui et al. (Matsui et al. 2008) , where a higher level of allergen was found to be associated with an increase in moisture levels. The results of Fereidouni et al. (Fereidouni et al. 2013) confirm that house dust mites in Iran are limited to the coastal areas of the Caspian Sea, while in other areas dust mites do not grow well due to seasonal changes in temperature and humidity. These results are consistent with the conclusion of our research. Moreover, the breeding rate of dust mites in an air conditioning system is affected by the cleaning frequency of the screens. A longer indoor ventilation time was negatively correlated with the breeding rate of dust mites.

A dust mite allergen concentration of 2 μg/g is considered the sensitization threshold for allergic diseases (Johnston et al. 2018) , and dust mite concentrations exceeding 10 μg/g may be a risk factor for asthma (Shafique et al., 2018). Research on environmental factors and allergies by Yadav et al. (Yadav et al., 2022) showed that dust mites are the main indoor pollutants that cause allergic symptoms. In this study, only two samples were detected for Der f 1 allergen and one sample for Der p 1 allergen concentration exceeding the threshold concentration (≥ 2.0 µg/g dust). Many studies have shown that family history can be one of the risk factors for the development of allergies. For instance, Chiesa et al. (Chiesa Fuxench, 2017) described that the risks of developing atopic dermatitis can be greater in anyone with a positive family history of atopic or allergic disease in either parent. Family history was quite positive in patients with childhood asthma and allergic rhinitis and common aeroallergens including HDMs, cats, and Alternaria were common in young children, while animal allergens were common in older children. Our observation is consistent with the results described above (Zahraldin et al. 2021) . Our observation is consistent with the results described above. In our research, we performed a questionnaire survey on the subjects regarding the family history of rhinitis, asthma, conjunctivitis, and recurrent eczema for risk factors affecting allergies. Most respondents reported exacerbated allergic reactions during the period of air conditioner use. Although many interviewees reported experiencing some allergic symptoms, they failed to undergo allergen tests, such as skin prick tests and blood IgE measurements, in professional medical institutions, which makes it difficult to clarify what allergen should be responsible for the allergic reaction. It is recommended that this part of the population perform relevant experiments to identify allergens and reduce the occurrence of allergic diseases by reducing exposure to allergens. Hence, individuals with an allergy should regularly replace their air conditioning filters, curtains, carpets, and mattresses and use proper personal protective equipment such as gloves and face masks in an allergen environment.

## Conclusion

In summary, we successfully determined the dust pollution status in the air conditioning system and the infection density of dust mites in dust samples from civilian houses, schools and hotel rooms throughout Anhui Province, China. We found that dust mite infection arising from indoor dust varied to a certain degree in geographical locations, quality of indoor hygiene and environment, such as temperature and humidity. These findings suggest that regular maintenance of indoor hygiene and cleaning of air conditioning filters can reduce the risks of exposure to indoor allergens.

## Data Availability

All the data analyzed during this study are included in this article.
